# Large-scale generation of *in silico* based spectral libraries to annotate dark chemical space features in non-target analysis

**DOI:** 10.1007/s00216-025-06034-4

**Published:** 2025-09-02

**Authors:** Emil Egede Frøkjær, Martin Hansen

**Affiliations:** Environmental Forensics and Metabolomics Laboratory, Department of Environmental and Resource Engineering, Technical University of Denmark, Bygningstorvet, Building 115, Kongens Lyngby, 2800 Hovedstaden Denmark

**Keywords:** C2MS CFM-ID, Groundwater, Non-target analysis, Mass spectrometry, Annotation

## Abstract

**Abstract:**

In this study, we develop and present an open-access LC-electrospray-HRMS/MS forward *in silico* fragmentation spectral library, based on the NORMAN Suspect List Exchange containing 120,514 chemicals, that can be used for level 3 annotations to support elucidation of the dark molecular features detected in environmental, exposomic, food safety, and forensic investigations. Using these forward generated *in silico* spectral libraries, several pollutants previously unreported in non-targeted workflows were discovered in groundwater for the first time through retrospective non-targeted screening analysis. Among these are xenobiotics such as hexafluoroacetone, hexazinone metabolites A, B, and C, and transformation products of triflusulfuron, fluazifop-butyl, triallate, and propiconazole. The generated *in silico* spectral libraries are freely available at https://doi.org/10.5281/zenodo.14854025.

**Graphical abstract:**

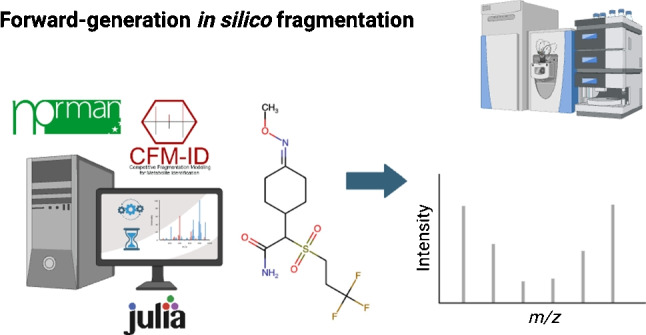

**Supplementary Information:**

The online version contains supplementary material available at 10.1007/s00216-025-06034-4.

## Introduction

Non-target analysis and suspect screening analysis (NTA) are powerful tools used in the elucidation of unknown chemicals in environmental [1–11], exposome [12, 13], food safety [14, 15], and forensic investigations [16, 17]. In NTA, samples or sample extracts are typically recorded using high-resolution mass spectrometry platforms hyphenated with liquid or gas chromatography separation techniques (LC-HRMS and GC-HRMS). In GC-HRMS, a hard ionization (electron impact) is most often used to generate a mass spectrum containing the pseudo-molecular ion and its fragments. In LC-HRMS, however, a soft ionization, electrospray, is typically used in combination with collision-induced tandem mass spectrometry to generate a fragmentation spectrum (MS2) of isolated precursor ions (MS1). Even though data processing pipelines and analytical platforms are under constant development, these methodologies often only manage to annotate a few percent of the total detected molecular feature space [18], as annotation of compound identities often relies on the presence of a given compound in reference spectral fragmentation libraries [19] or through the use of masslists or *in silico* tools [20–22]. And even then, the annotation confidence for the majority of annotations rarely exceeds that of tentatively suggested structures (levels 3–4 according to the Schymanski scale [23]). This especially poses a challenge when reporting findings to regulatory institutions and authorities as they often refute low-confidence annotations. Subsequently, most lower level annotations tend to get ignored in favor of higher confidence annotations, where these are then confirmed (level 1) or rejected through the purchasing of analytical reference standards. However, the scientific and regulative community risk losing valuable information hidden outside the chemical space contained by experimental reference spectra. And if one relies solely on high-confidence annotations, NTA pipelines become practically indistinguishable from traditional targeted analysis with circular identification workflows with little to no new discoveries. If scientists are to continuously push NTA methodologies forward and increase the identifiable space [24–26], it is vital to continue the development and utilization of computational tools [27, 28]. Many initiatives are made to annotate the “dark” or unannotated chemical space. A frequently used methodology is the generation and implementation of wide-scope, but narrowly aimed, suspect lists. One such list is the NORMAN SusDat 2024 [29] list, containing 120,514 compounds of particular interest for the environment. The implementation of suspect lists improves the annotation space greatly—at the cost of annotation confidence, as most annotations are then made solely from a matching m/z value. However, by combination with *in silico* fragmentation techniques, suspect lists could be used for both MS1 and MS2 compound matching at a potential confidence level of 3 with the possibility to identify MS2 spectra relevance where no reference match exists either due to lack of data entries or chemical unavailability of reference compounds.

There are two main ways of performing *in silico* fragmentation for the annotation of (unknown) compounds: the reverse approach, also referred to as spectrum-to-compound (MS2C), and the forward approach (compound-to-spectrum (C2MS)) [22]. In MS2C, an experimental fragmentation spectrum is recorded and compared to a database of known structures in order to rank the most probably structure based on matching fragmentation pattern. Such methods are well described in literature and can be implemented through various tools like CFM-ID [22], FISh [30], MetFrag [20], DarkNPS [31], MS-Finder [32, 33], CSI:FingerID [21, 34], and the *de novo* approach MSNovelist [35], where structural elucidation can be extended to completely unknown structures. Reverse fragmentation tools are thus useful in finding structural candidates for spectra where no experimental reference spectra are matched and have been shown to widen the identifiable space of novel compounds [36–40]. The implementation of these tools is however not always straight forward and often requires *some* knowledge of coding to avoid doing manual search and curation for potentially thousands of spectral candidates. Instead, the forward fragmentation approach (C2MS) can be implemented to predict spectra from known structures found in, for example, suspect lists. This is especially beneficial as C2MS can be used to improve the confidence of suspect screening by generation of predicted spectra. As these predicted spectra can be used to perform spectral similarity scoring in the same way as with experimental spectral libraries (by ranking similarity by, for example, cosine score), the implementation of *in silico* libraries—once generated—requires little to no knowledge of coding to implement. Examples of the forward *in silico* approach have been widely explored in literature with models such as LipidBlast [41], MyCompoundID [42], CFM-ID [22], GrAFF-MS [43], NPS-MS [44], MassKG [45], and SingleFrag [46]. Of these, CFM-ID is especially well founded for the generation of predicted fragmentation libraries. Using SMILES [47], *in silico* libraries have been made for the entire DSSTox database [48, 49]. CFM-ID v. 2.0 has additionally been used as a forward prediction tool to generate an *in silico* library of the NORMAN SusDat knowledgebase [50]. Likewise, CFM-ID has been used to generate an *in silico* library of predicted biotic and abiotic transformation products of PFAS [51] with this library being freely available, allowing for a great boost in identification rates within the PFAS community.

Both of these *in silico* techniques complement each other. While reverse screening can be associated with a non-targeted methodology, forward screening shares characteristics with that of ordinary suspect screening. As such, pregenerated C2MS libraries can be used as high-confidence screening tools in both ordinary and retrospective suspect screening analysis to assist in the discovery of novel and potentially hazardous chemicals [36, 52, 53]. With community effort, these findings can then be shared and integrated into public MS2 repositories for improvements in future identification workflows. Another utility of forward *in silico* libraries is the ability to easily include retention time values, if known, as many processing software allow for a retention time windows threshold during both spectral matching and masslist searches. Thus, an added layer of confidence can be attained for compound annotations (by easier filtration of false positives) if measured retention times do not fall outside the desired range. For unknown retention times of known structures, these can be predicted (within a certain accuracy) using some of the many available prediction tools [54–58].

As such, it is the opinion of the authors of this work that *in silico* methodologies constitute an essential part in NTA workflows and should not be omitted, as they are necessary to fill the information gap between the detectable and identifiable chemical space. While it is understood that most *in silico* tools rely on the user’s ability to program and integrate solutions into specific applications, this should not prevent some researches from fully utilizing these techniques. We therefore present the complete NORMAN SusDat suspect list (as of version 2024) prepared as *in silico* generated spectral libraries, ready for direct use in open-access software such as MZmine [59] and MS-DIAL [60], as well as commercial software such as Compound Discoverer, to support the community in the discovery of novel compounds in NTA studies.

## Methods

### Software and packages

Several software and software packages were used throughout this study: *Excel 365* (Microsoft) was used for quick data visualization and prioritization; *CFM-ID* 4.4.7 [22, 61–65] was used to perform *in silico* calculations of compound SMILES; *Docker Desktop* 4.28.0.0 (https://www.docker.com/) was used to connect to the CFM-ID container for non-web-based batch processing; *PowerShell* (Microsoft) was used to call command lines through the CFM-ID Docker image for batch processing; *Julia* 1.10.2 [66] was used for data clean-up of CFM-ID outputs, filling of metadata, package integration for structural clean-up, and for generation of figures used throughout; the *RDKit* package (version 2024.09.4) [67] was used for structural clean-up of SMILES and to obtain compound metadata; *PugRest API* (https://pubchem.ncbi.nlm.nih.gov/docs/pug-rest) [68] was used to obtain information of missing SMILES; *mzVault* 2.3 (Thermo Scientific) was used for conversion of.msp outputs to.db format; *DB Browser for SQLite* 3.12.2 (https://sqlitebrowser.org/) was used to perform SQLite queries on generated databases for fast structural assignment; *Matlab* R2024a [69] was used for merging of database duplicate entries using Database Explorer integration [70]; *MZmine* 4.3 [59] was used for feature detection in a non-targeted LC-HRMS dataset; *Chemical Sketch Tool* (https://www.rcsb.org/chemical-sketch) was used for drawing of chemical structures.

### Masslist clean-up

The NORMAN SusDat (version 2024) suspect list was downloaded as.xlsx format from https://www.norman-network.com/nds/SLE/. This version contains information of 120,514 unique compounds present in NORMAN suspect list exchange lists S001-S111. Of these, 110,402 (91.6% of the original list) compounds had compatible SMILES. The remaining missing SMILES entries were mined from PubChem [71] using the PugRest API [68] through a Matlab script. Thus, an additional 2997 SMILES were obtained through either names, CAS, InChI, or InChIKey, to reach a total of 113,399 (94.1%) substances with computation ready SMILES (cf. SI-1). Before *in silico* predictions, RDKit [67] was used to clean-up the SMILES structures by removal of salts and neutralization of the structure. After this step, 330 structures were still in their salted form (i.e., containing “.” in the SMILES string) and were removed from the dataset. Then, 67 compounds with masses less than 40 Da were removed, as well as 1487 compounds with masses higher than 1000 Da—including all 336 compounds with more than 200 atoms (the current limitation for CFM-ID calculations). Lastly, 316 compounds with charge $\ge$2 were removed, as CFM-ID only supports calculation of single-charged species. As some de-salted structures might be identical, identical SMILES were merged by string comparison of canonical SMILES, removing replicates, resulting in a final number of 93,590 unique compounds. SMILES representing various stereoisomers, e.g., were kept due to their different chemical representation, though these would provide (near-)identical predicted MS/MS spectra. The compounds were then divided into three groups: negatively charged compounds (50) for [M+e]^-^ predictions, positively charged compounds (2,536) for [M-e]^+^ predictions, and 91,004 neutral compounds for [M+H]^+^ and [M-H]^-^ predictions, respectively. All other adduct types (e.g., ammonium and sodium) are not supported by CFM-ID calculations and were therefore omitted from this study.

### CFM-ID 4.4.7 calculations

*In silico* calculations of SMILES were performed in batch mode using CFM-ID 4.4.7 [22] through the use of PowerShell and connection to the CFM-ID Docker container. CFM-ID provides a predicted spectrum for 10 eV, 20 eV, and 40 eV for each SMILES entry, and as such, it calculated around 550,000 spectra, which took around 10 days on a Thinkpad T16 AMD Gen 2 (AMD Ryzen 7 Pro 7840U / 3.3–5.1 GHz / 16 MB Cache, 32 GB LPDDR5X DRAM). The resulting output file formats were .msp (NIST) and contained information of compound ID, SMILES, collision energy, the number of fragments, and m/z and abundance of each fragment.

### NIST format

Metadata of the output .msp-files were filled in using Julia [66] and RDKit [67] to obtain InChI, InChIKey, adduct type, ionization mode, corrected collision energies, formula, and precursor mass for each entry. This allowed for easy integration into open-access data processing software such as MZmine [59]. Compound names were kept as the SusDat ID to avoid special characters while still allowing for easy cross-referencing with the NORMAN Suspect List Exchange knowledgebase (https://www.norman-network.com/nds/susdat/).

### Database format

For integration into the commercial software Compound Discoverer (Thermo Scientific), additional steps are required to convert the .msp file format into a cleaned database (.db format). For this, Julia, mzVault, Matlab, and DB Browser for SQLite were used. First, as part of the .msp output clean-up procedure described above, the Julia script also corrects for potential inconsistencies between defined peak number and actual peak number if present. Afterwards, mzVault was used to import and convert the .msp into a .db format. The resulting database file thereby contained a compound entry per spectrum (thus three entries per compound—one for each collision energy) with lacking structural information. mzVault could in principle be used to mine both metadata—including molecular structures—and to merge duplicates. However, this is done by mining the ChemSpider [72] knowledgebase, which is both time-consuming and can result in still missing—or even incorrect—annotations. Another challenge was—due to the size of the databases (over 250,000 entries in each)—that merging and metadata search within the mzVault software was not easily resolved without experiencing stability and run-time issues. Instead, DB Browser for SQLite in conjunction with Julia and Matlab was used to quickly (in less than 10 min) merge duplicate compound entries and mine and insert molecular structures (SDF-format) using SQLite queries. The cleaned databases could thus be used directly with Compound Discoverer, containing all necessary (structural) metadata for optimal identification workflows with respect to both ease-of-use and accuracy.

### Feature detection and spectral matching

To explore and to evaluate the quality of the generated *in silico* libraries, a feature detection workflow using MZmine [59] was performed on a dataset of 81 ground water samples previously analyzed with a nanoLC-Orbitrap Q Exactive HF (240K) recorded in ddMS2-mode using 20, 70, and 120 NCE in stepped collision energies, described in detail elsewhere [73]. Peaks were identified using a minimum intensity threshold of 1E6 and a mass accuracy threshold of 5 ppm. Following smoothing [74], feature resolution, alignment across samples (±0.50 min), keep MS2-only, isotope, and duplicate filtering, gap filling, ion identity networking [75], and blank filtration (S/B > 10), detected features were screened towards either experimental spectral libraries (described below) or the generated *in silico* libraries. Spectral matching was done using a precursor m/z tolerance of 3 ppm, fragment m/z tolerance of 5 ppm, and unweighted cosine similarity scoring, defined in Eq. 1, where ${\textbf {v}}$ and ${\textbf {u}}$ are intensity vectors of the experimental and reference spectra, respectively, with a minimum score of 0.1 and minimum required number of fragments of 2—including the precursor ion. Though several modifications (weighting towards intensities and/or m/z) exist for calculating the cosine similarity, the unweighted algorithm was used for the most general representation of similarity scoring.

1 $$\begin{aligned} \text {cos}\theta =\frac{{\textbf {v}}\cdot {\textbf {u}}}{\left| {\textbf {v}}\right| \cdot \left| {\textbf {u}}\right| } \end{aligned}$$

Unmatched reference and experimental signals were kept but matched to zero. Results were then manually investigated to filter out potential false positives. The lenient spectral search parameters were implemented to allow for more matches in the initial data processing by ensuring the inclusion of compounds with either little fragmentation or with spectrum predictions of sub-optimal quality. This step was important to prevent the loss of many interesting suspects as empirical data suggested that spectra measured on the Orbitrap system were heavily affected by both chimeric and/or matrix-related interference. Since CFM-ID is trained on (and predicts) spectra obtained on QTOF analyzers, small discrepancies are expected in the spectral similarity scores between these two analyzer architectures (TOF and Orbitrap). Exact MZmine-processing parameters can be found in the batch file attached with the Supporting Information.

### Experimental spectral libraries

A total of 1,543,129 high-resolution reference spectra were obtained through various open-access spectral databases: *MS-DIAL* [60], version 19, downloaded August 8, 2024, 367,674 spectra. *MSnLib* [76], downloaded April 11, 2024, 85,382 spectra. *Massbank of North America (MoNA)* (https://mona.fiehnlab.ucdavis.edu/), downloaded July 11, 2024, 148,546 spectra. *Massbank EU* [77], downloaded June 5, 2024, 117,283 spectra. *GNPS* [78], a public collection of user-curated spectral databases, downloaded November 1, 2024, 816,675 spectra. *HighResNPS*, a spectral library containing entries of new psychoactive substances [79], downloaded April 28, 2023, 3280 spectra. *Folberth et al.* [80], a library of endogenous metabolite, downloaded October 30, 2024, 4159 spectra. *Wang et al.* [81], a spectral library of polyfluoroalkyl substances (PFAS), downloaded July 9, 2024, 130 spectra.

## Results and discussion

### Validation

**Fig. 1:**
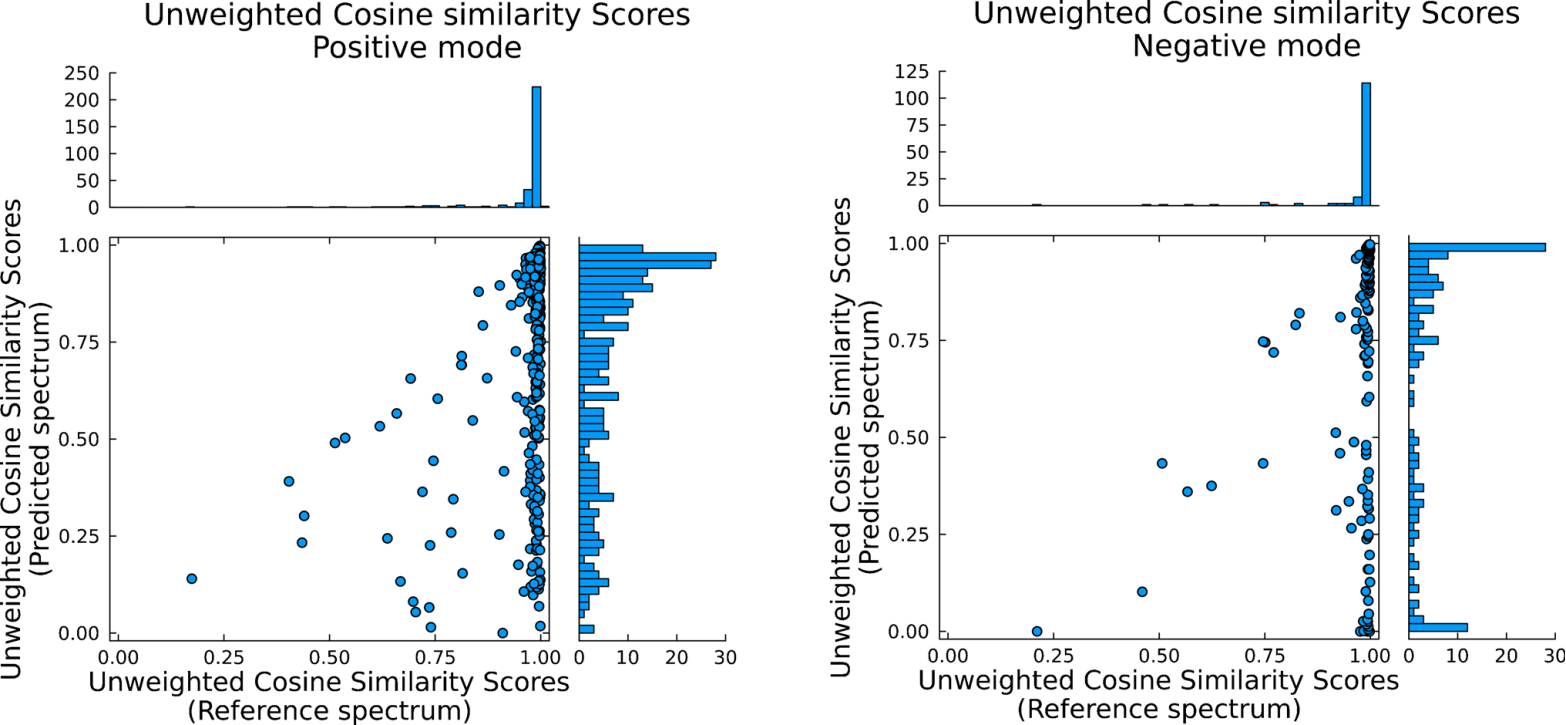
Unweighted cosine similarity scores between predicted spectra (*y*-axes) and reference spectra (*x*-axes) for 344 analytical standards (301 in positive mode, 139 in negative mode) spiked in matrix and recorded in positive and negative electrospray ionization modes, respectively

**Fig. 2:**
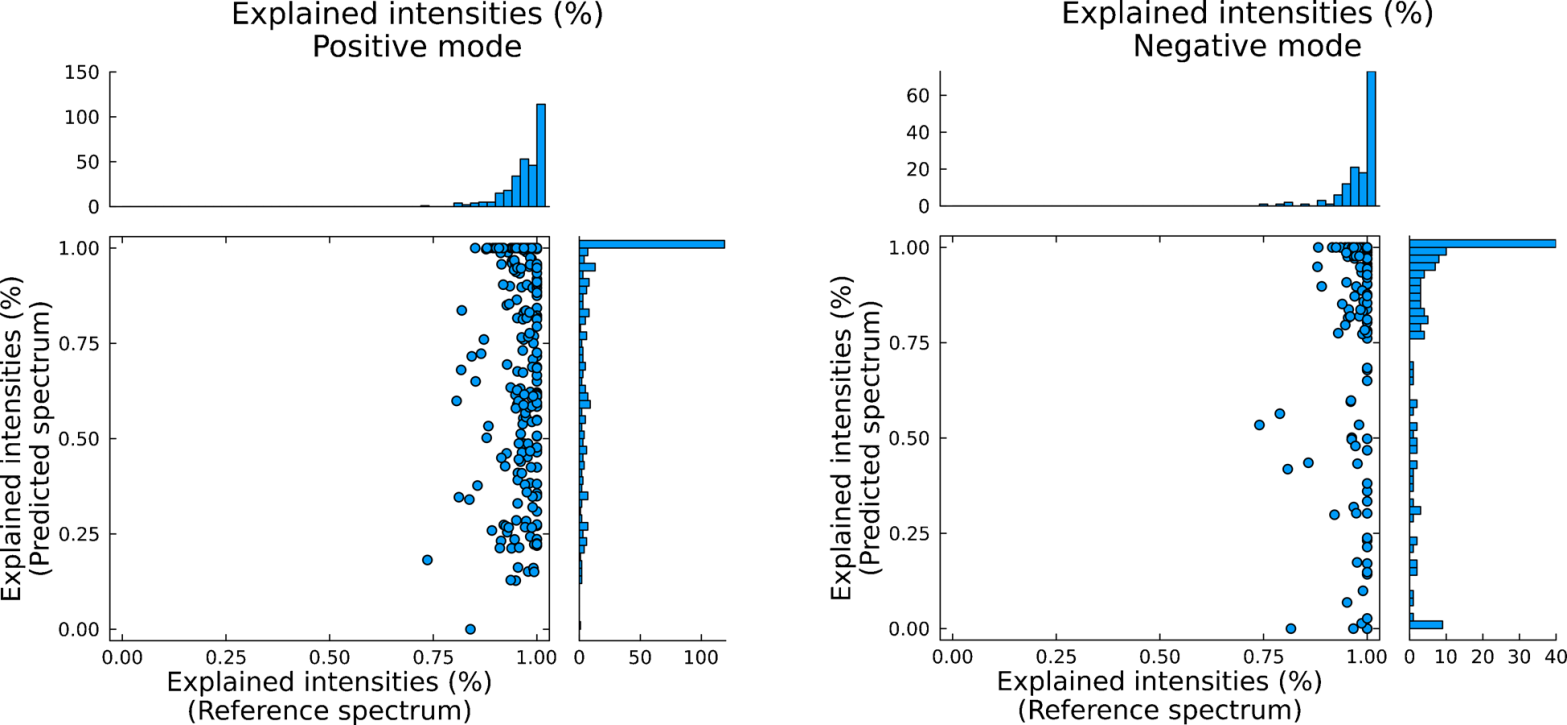
Explained intensity between predicted spectra (*y*-axes) and reference spectra (*x*-axes) for 344 analytical standards (301 in positive mode, 139 in negative mode) spiked in matrix and recorded in positive and negative electrospray ionization modes, respectively

**Fig. 3:**
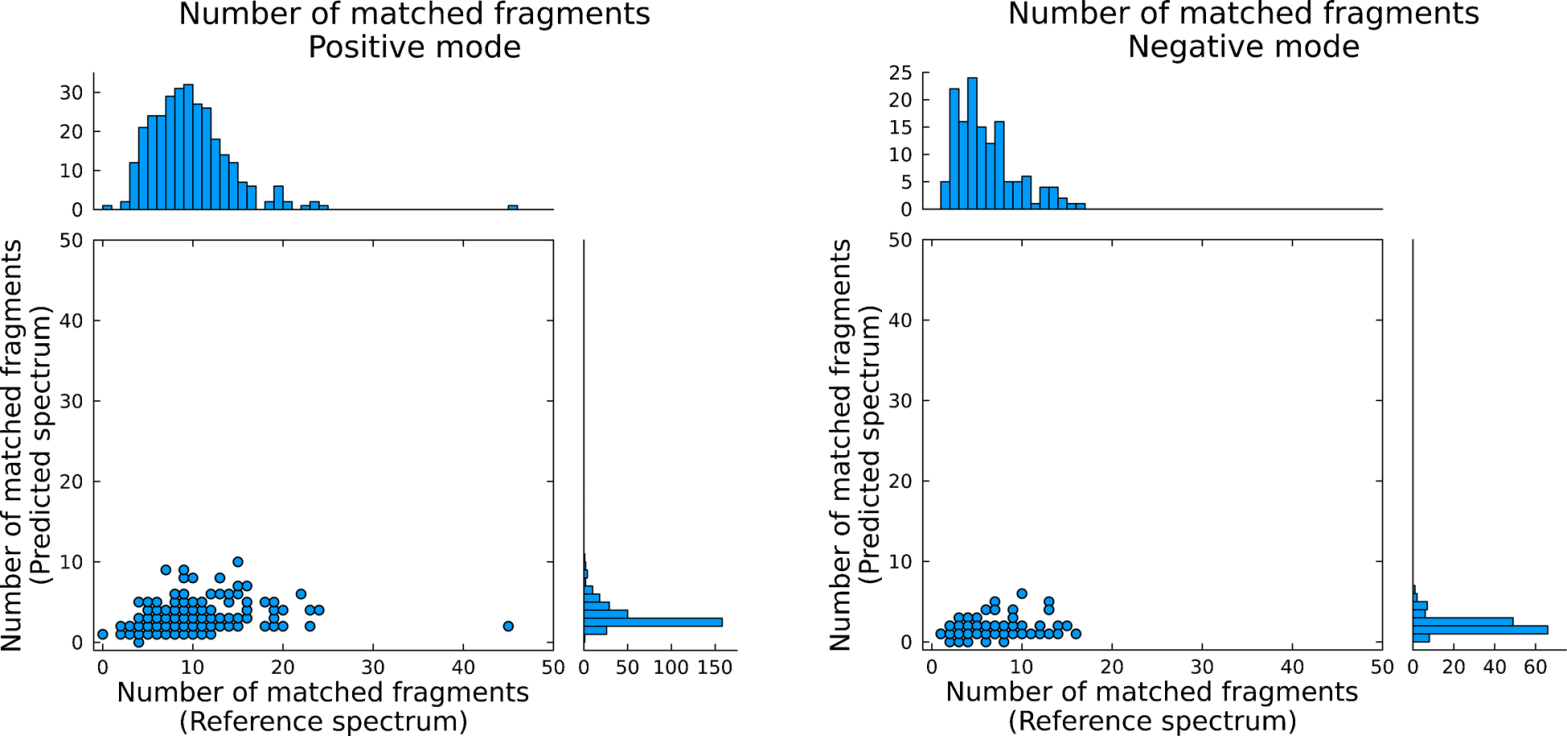
Number of matched fragments between predicted spectra (*y*-axes) and reference spectra (*x*-axes) for 344 analytical standards (301 in positive mode, 139 in negative mode) spiked in matrix and recorded in positive and negative electrospray ionization modes, respectively

To assess the quality of the *in silico*-based annotations, 344 chemical standards (cf. SI-3) were spiked (100 ng of each compound) into a 1 L groundwater sample—prior to sample preparation—for an anticipated final concentration of 100 ng/L. These standards were then annotated in matrix through MZmine (301 and 139 in positive and negative modes, respectively) from in-house reference spectra that had previously been recorded in pure solvent at similar acquisition parameters. Spectral similarities were then compared to those of the *in silico* in regards to unweighted cosine scores, explained intensity, and the number of matched fragments shown in Figs. 1, 2, and 3.

Comparing the experimentally measured spectra of the 344 compounds with those recorded in pure solvent (Fig. 1), the 10th percentile (i.e., 90% of data values were above this value) of cosine similarity scores were 0.903 and 0.929 for positive and negative modes, respectively. In contrast to this, the 10th percentile scores were only 0.222 and 0.03, respectively, when compared to the *in silico* generated spectra. Of the 344 validation compounds, 107 were part of the original training data for the CFM-ID prediction model (see SI-2). The spectral similarity score is thus significantly lower for the predicted spectra—and especially for spectra predicted for negative mode—than when comparing towards experimentally recorded reference spectra. This means that it can be difficult to define a specific similarity score threshold for CFM-ID predicted spectra, which impacts the number of false/true positive annotations obtained in a classic non-targeted data pipeline. To obtain a better understanding of the relationship between similarity score and experimental and predicted spectra, looking deeper into the similarity scores for the experimental reference spectra reveals that less than 7% of the recorded spectra had spectral similarity scores below 0.8 and only around 4% less than 0.7. Thus, more than 95% of similarity scores based on experimental reference spectra were above 0.7 (the minimum required value that, according to the community consensus, is required for an appropriate compound annotation [19, 78].) Still, even under identical acquisition parameters, a small number of compounds (12 and 5 in positive and negative modes, respectively) would be rejected based solely on their similarity scores being below the threshold of 0.7. Applying the same threshold to filter through similarity scores of predicted spectra would reject nearly 40% of the true positive annotations (126 and 54, respectively), clearly indicating a need for adjustment in traditional scoring thresholds when implementing predicted spectra.

Looking at the other scoring metrics, specifically the explained intensity scores (Fig. 2), only 3 experimental entries had scores between 0.7 and 0.8, with the remaining 341 entries having an explained intensity score above 0.8—and more than 90% of those having a cosine score above 0.9. There is therefore a better correlation between true positive annotations and the explained intensity score—and a very high correlation when accounting for both cosine and explained intensity scores. Compared to the scores for the predicted spectra, the values are again significantly worse than for the experimental reference spectra. More than 30% of explained intensities were below 0.7 for both positive and negative modes. In positive mode, 58% of predicted spectra had cosine scores > 0.7, with 74% > 0.5. Of those above 0.5, 90% had an explained intensity value above 0.5 as well. As such, reducing the required thresholds for both cosine similarity and explained intensity to, for example, 0.2 and 0.7, respectively, should account for the reduced values when using predicted spectra. With a higher threshold of explained intensity, the likelihood of false positives is reduced. Also worth noting is the amount of matched fragments (Fig. 3). A minimum value is in some software (e.g., MZmine) required before performing spectral search and is important in order to reduce the number of false positives. If not chosen carefully however, this value can easily filter out true positive candidates if set too high. From Fig. 3, it is seen that the 10th percentile was 4 and 2 for positive and negative modes for reference matches, but 2 and 1 for the *in silico* libraries. In fact, 48% of the predicted spectra in negative mode had only 1 matching fragment. Thus, when performing spectral scoring, it is important to consider the number of fragments to include. Allowing for a lower number of matched fragments (2 or 3) increases the amount of true positives, while the risk of false positives can be reduced by accounting for both the explained intensity, cosine similarity, and spectral purity. As such, it is recommended to keep predicted and experimental reference spectra in separate spectral repositories for better (and more correct) annotation rate and to adjust the threshold settings accordingly.

As less than 5% of cosine scores between the experimental and reference spectra of confirmed compounds were below 0.7, a threshold of this value seems ideal in regards to data filtration. When comparing experimental spectra with external experimental MS2 reference spectral libraries, a reduction in similarity scores is to be expected. This is in part due to possible impurities in the experimental spectra originating from matrix effects and isobaric and/or chimeric interference, and deviations between the analyzed and compared spectra with respect to mass accuracy, instrumentation/analyzer type, acquisition parameters, and sub-optimal fragmentation settings [82]. For predicted spectra, any discrepancy between measured and predicted fragment intensities can be assigned to the uncertainty of the specific prediction model.

If experimental information indicates a reduction in the quality and/or purity of either the experimental or reference spectrum, the likelihood of a low cosine similarity score increases. Some knowledge of spectral quality is thus recommended when evaluating the annotations as reliance on a single mathematical value is often insufficient to fully determine whether or not a compound annotation is true or false. Following this, it is acceptable to annotate compounds with cosine scores less than 0.7 if sufficient evidence can back up the claimed identity. This can be, for example, the explained intensity, knowledge of retention time indices [54] and the isotopic pattern at the MS1 level. Especially the explained intensity is important. This is because explained intensity is less affected by spectral impurities and, as such, better differentiates between low cosine scores caused by either non-similarity or matrix and/or chimeric induced experimental impurities. Thus, a better measure of spectral similarity is an evaluation based on *both* cosine score and explained intensity, where thresholds for these values could be set to 0.7 and 0.9, respectively, for improved rejection of false positives. And as long as strict thresholds of the explained intensity were maintained, it would even be possible to reduce cosine score thresholds to as low as 0.4, increasing the annotatable space at the cost of an increase in the number of false positives.

### Comparing *in silico* and experimental spectral libraries

The generated *in silico* libraries were evaluated by comparison with open-access experimental reference libraries on a groundwater dataset previously described and analyzed [73]. In negative mode, 3116 MS2-containing features were detected. Using the generated *in silico* libraries, 549 spectral matches were obtained at a cosine score $\ge$ 0.7, with an additional 586 matches between 0.1 and 0.7 and 1981 substances with scores < 0.1. In contrast, using experimental reference libraries, 261 annotations were obtained at a cosine $\ge$ 0.7, 461 matches between 0.1 and 0.7, and 2394 matches with scores < 0.1. In positive mode, 11,682 MS2-containing features were detected. A total of 1561 spectral matches were obtained from the *in silico* library at a cosine score $\ge$ 0.7, with 1846 matches between 0.1 and 0.7 and 8275 features remaining unannotated. Using experimental reference libraries, 755 annotations were found at cosine score $\ge$ 0.7, 1551 between 0.1 and 0.7, and 9376 at < 0.1. Thus, roughly twice as many annotations can be obtained in groundwater samples by using the generated *in silico* libraries compared to using only reference spectra. Following this, a significant amount of the hits (with a score above 0.7) could be attributed solely to the *in silico* libraries.

With a total annotated coverage of 606 out of 3116 (19%) in negative mode and 1764 out of 11,682 (15%) in positive mode, only around 10% of this can be uniquely attributed to the use of reference libraries (57 and 203 unique hits, respectively). The remaining 90% are either uniquely attributed to *in silico* libraries or are in common between the two library types (cf. SI-4). Thus, the annotatable space is greatly improved through the use of *in silico*-based fragmentation libraries. It should be stated that overlapping hits between reference and *in silico* hits in this context do not necessarily correspond to identical annotations but simply if a detected feature was assigned an annotation during both the library searches. Considering that many *in silico* entries are not found in reference libraries, the overlapping annotations likely differ. Still, using *in silico* libraries to assist with compound annotation more than doubled the number of annotations obtained compared to using only experimental reference libraries.

**Table 1:** Overview of tentatively identified and confirmed compounds found in the groundwater samples using the *in silico* generated library

Compound	Type	Mode	cos$\theta ^a$	cos$\theta ^b$	Level
Hexafluoropropane-2,2-diol* AKVXSYUWYXOLMY-UHFFFAOYSA-N	PFAS	-	0.247	0.984	1
Propiconazole TP SYN 547889 NUAGPTNJVDKMOR-UHFFFAOYSA-N	Fungicide TP	-	0.327	0.995	1
Triallate TP TCPSA GLDBPELSAPUAFU-UHFFFAOYSA-N	Herbicide TP	-	0.381	0.997	1
Isodrin TP1 BMZDODNMJBZLAB-UHFFFAOYSA-N	Insecticide TP	-	0.624		3
Para-hydroxy triphenyl phospate NOPNBQOZUKISRP-UHFFFAOYSA-N	Flame retardant TP	-	0.847		3
2,4-Dichlorophenylacetic acid GXMWLJKTGBZMBH-UHFFFAOYSA-N	Herbicide TP	-	0.992		3
Fluazifop-butyl TP CGA142110 BYRJSCNPUHYZQE-UHFFFAOYSA-N	Herbicide TP	+ (-)	0.992	0.999	1
Hexazinone TP A ASCFMHBRJVKECO-UHFFFAOYSA-N	Herbicide TP	+	0.729		3
Hexazinone TP B YCIQIUHJVFRTTB-UHFFFAOYSA-N	Herbicide TP	+	0.711		3
Hexazinone TP C YLNFKJPRYUIXTG-UHFFFAOYSA-N	Herbicide TP	+	0.813		3
Triflusulfuron-methyl TP IN-M7222 HJZAYYJWOHOQSM-UHFFFAOYSA-N	Herbicide TP	+	0.946	0.984	1
5-Chloroisatoic anhydride MYQFJMYJVJRSGP-UHFFFAOYSA-N	Industrial	+	0.992		3

$^{a,b}$Similarity score for *in silico* and recorded reference spectrum, respectively. *Identified as hexafluoropropane-2,2-diol and confirmed by aqueous solution of hexafluoroacetone hydrate

As the applied mass threshold of the spectral library search was 3 ppm, this could exclude potential candidates present in the reference databases where lower MS1 resolution (compared to 240,000) was used. Despite being correct annotations, these entries might have mass deviations greater than 3 ppm compared to the recorded data. As such, the number of annotations achieved from the reference spectra might be lower than the highest possible amount and could have been increased with increasing mass tolerance.

### Identification of compounds not present in reference libraries

**Fig. 4:**
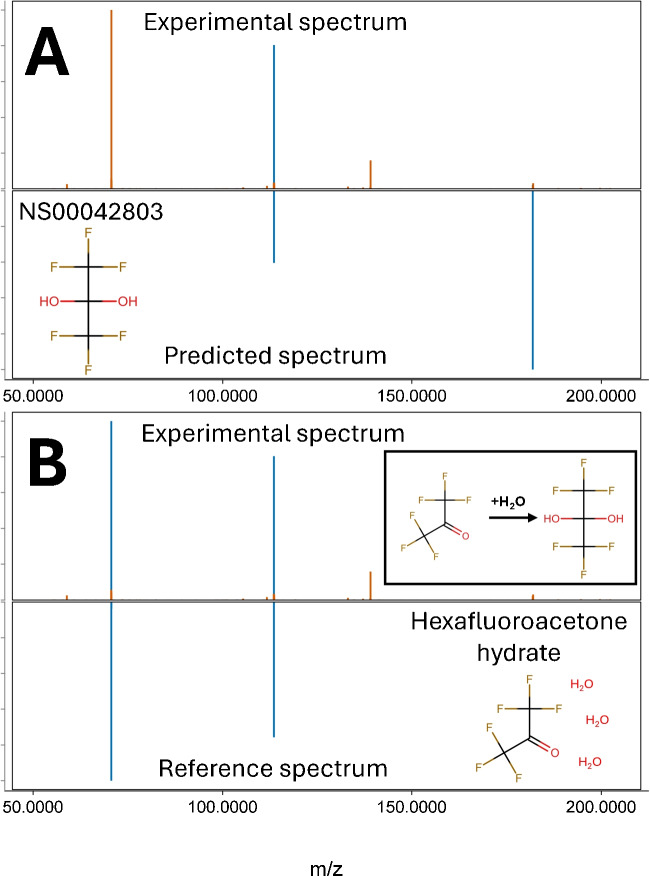
Spectral comparison of experimental, predicted, and reference fragmentation spectra for feature m/z 182.9887 (identified as hexafluoroacetone) recorded in negative mode. **A** (Top) Experimental fragmentation spectrum for m/z 182.9887. (Bottom) predicted *in silico* spectrum for hexafluoropropane-2,2-diol (NS00042803) with 2 matching fragments and a cosine similarity score of 0.247. **B** (Top) Comparison of the same experimental spectrum for m/z 182.9887 matched with (bottom) a reference standard of hexafluoroacetone hydrate with a cosine score of 0.984. Insert shows the presumed reaction between hexafluoroacetone and water producing hexafluoropropane-2,2-diol

Using the generated *in silico* libraries, several new pesticides transformation products (TP) were discovered in the groundwater samples (Table 1). Despite having cosine scores between 0.2 and 0.4, some were confirmed at level 1 using reference standards: triallate TP TCPSA (NS00008158), propiconazole TP SYN 547889 (NS00114332) (previously identified in Danish surface waters [83]), fluazifop-butyl TP CGA142110 (NS00067405), and triflusulfuron-methyl TP IN-M7222 (NS00067847). The reason for the low similarity scores could be due to none of the identified compounds being captured well by the CFM-ID predictions model, as they were not part of the original training set of the model. A PFAS previously unreported, but recently discovered in drinking water [84], was also detected and confirmed at level 1: The compound hexafluoropropane-2,2-diol (NS00042803) was detected at m/z 182.9887 and confirmed by a reference standard of hexafluoroacetone (HFA) hydrate (Fig. 4). When dissolved in aqueous environment, this substance readily reacts with the water to create the geminal diol compound ($K_\text {eq} = 1.2\cdot 10^6 M^{-1}$) that can be detected in the samples [85, 86]. Another HFA adduct was detected at m/z 228.9941 corresponding to the [M+CH$_2$O$_2$-H]$^-$ ion. Characteristic fragments of m/z 68.9958 (CF$_3^-$) and 112.9856 (CF$_3$COO$^-$) were seen present in both spectra. It was not possible to obtain a reference standard for hexafluoropropane-2,2-diol, and as such, it had to be confirmed indirectly through HFA. As a consequence, it is currently not possible to determine whether the detected PFAS is indeed hexafluoropropane-2,2-diol or a transformation product of HFA.

From these observations, it is clear that true positive predicted spectra can be found for even very low cosine scores. The evaluation of cosine similarity scores is entirely data-driven and can yield widely different results based on the chosen similarity algorithm and weights [87]. This is significant to consider, as results from this study show that CFM-ID predictions can provide true positive annotations on an Orbitrap system at thresholds as low as 0.25. Given that this threshold is generally not feasible for untargeted screening studies, as a majority of matches would be expected as false positives, it does however highlight the necessity of critically assessing the quality of spectral data in both reference and experimental libraries. The spectral similarity threshold should be defined based on empirical evidence of the instrumental settings, sample complexity, and library quality and can, in some scenarios, be lowered to allow for more annotations. Irregardless of the threshold chosen, the resulting data requires some type of curation step to prevent false positives. Following this, it is then up to the scientist(s) to decide whether an increase in the identifiable space is worth the time spent on data curation. To reduce the subjectivity related to manual curation, the identification point (IP) system was proposed as an objective way to report annotation confidence based on various spectral metrics [88].

**Fig. 5:**
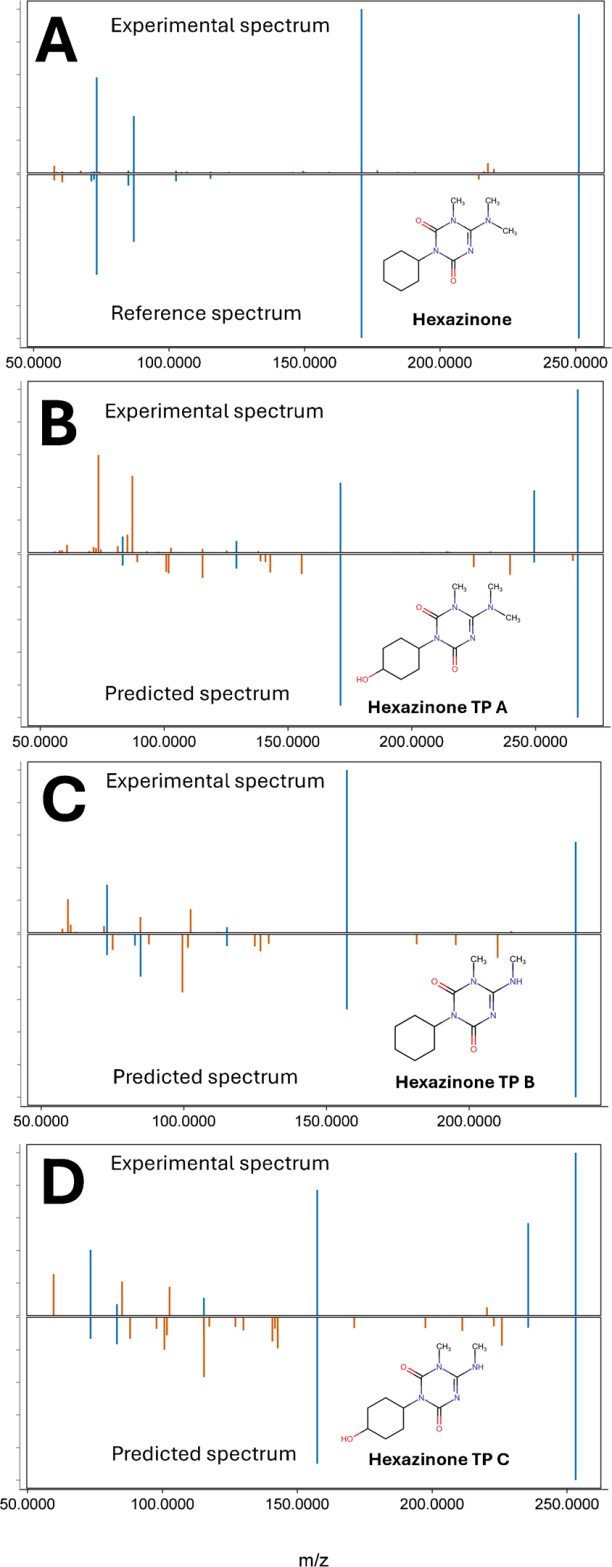
Spectral matches for hexazinone and three transformation products. **A** (Top) Comparison of an experimental spectrum at m/z 253.1659 in positive mode with (bottom) a reference standard match of hexazinone with a cosine score similarity of 0.996 and matching retention time at 20.06 min. **B** (Top) Experimental spectrum at m/z 269.1605 and (bottom) predicted spectrum for hexazinone metabolite A with a cosine score of 0.729. **C** (Top) Experimental spectrum at m/z 239.1501 and (bottom) predicted spectrum for hexazinone metabolite B with a cosine score of 0.711. **D** (Top) Experimental spectrum at m/z 255.1450 and (bottom) predicted spectrum for hexazinone metabolite C with a cosine score of 0.813

In two samples, the herbicide hexazinone was identified at level 1 using a reference standard. In the same two samples, three other hexazinone transformation products (hexazinone TP A, hexazinone TP B, and hexazinone TP C) were annotated (at level 3) from the generated *in silico* libraries (Fig. 5). Hexazinone and its metabolites have previously been detected in groundwater [89] and are as such not an unexpected find. However, none of these three metabolites are present in the used reference spectral libraries and thus have likely been overlooked from traditional NTA workflows.

Several other compounds of interest were also detected when using the *in silico* libraries (Table 1), e.g., isodrin TP1 (NS00102650), para-hydroxy triphenyl phosphate (NS00115818), 2,4-dichlorophenylacetic acid (NS00008251), and 5-chloroisatoic anhydride (NS00031689). Notably, none of these compounds were present in the training set for the CFM-ID model. A full list of annotations and training compounds for the CFM-ID model can be found in the Supporting Information (SI-2).

From these findings, the strength of forward (C2MS) *in silico* fragmentation is showcased. Other studies saw an improvement in screening and annotation accuracy [90, 91] when using lists of cleaned SMILES, neutralized and de-salted molecules, and calculation of proper precursor masses based on the molecular charge. A similar clean-up step was performed on every entry in this study in order to improve the prediction quality, expanding on the more than 70,000 already present entries in the NORMAN SusDat containing MS-ready (i.e., neutralized) SMILES with corresponding formulas and monoisotopic masses. Despite this, some limitations are still found in the presented forward *in silico* approach. For one, only a limited amount of adducts—namely (de-)protonation and natively charged—are supported by the current CFM-ID algorithm. Therefore, compounds that ionize without (de)protonation, such as for Na$^+$ or NH$_{4}^+$ adducts, or dimer or double charged species, such as for mycotoxins [92], some pesticides and pesticide transformation products [93], and peptides [94], would remain unidentified. Since CFM-ID predicts QTOF spectra at 10, 20, and 40 eV [40], a need for translation onto other systems (or collision energies) is recommended in order to reduce false-positive matches. In the case of this study, the predicted spectra at 20 eV seemed most compatible with the recorded data.

As a concluding remark, around 18% and 13% of detected features with associated MS2 spectra could be annotated by the generated *in silico* libraries with a cosine score >0.7 in negative and positive modes, respectively. Compared to the usage of experimental libraries (>1.5 M spectra), where 8% and 6% were annotated, the generated *in silico* libraries show great promise in the expansion of the identifiable space—as well as the confident discovery of new contaminants—in NTA workflows. Compound identification rate increased by more than a factor of two with the implementation of *in silico*-based reference libraries. Together with suspect lists, forward *in silico* allows for easier prioritization and identification of relevant pollutants and can improve the candidate selection process when performing annotation verification through the purchase of reference standards. It further has the potential to support metabolite prediction tools such as BioTransformer [95, 96] or enviPath [97, 98] by the addition of spectral information to predicted structures. As with any NTA workflow, it is important to assess retention times of the annotated species to filter between structural candidates and to reduce false-positive annotations caused by in-source fragmentation [99, 100]. When retention times are known, they can be used as a strict threshold during feature annotation, filtering all unlikely structural candidates based on their elution profile. When retention times are unknown, prediction tools can be used to estimate retention times of features based on either proposed structures [54, 56, 58, 101] or directly from their MS2 profile [55, 57]. Annotation threshold settings can then be defined based on the prediction confidence of the model, e.g., $2\sigma \!$, assisting in the removal of false-positive candidates. More than 90,000 retention time indices have already been predicted and are available for the NORMAN SusDat List, readily translatable to system specific retention times through the use of overlapping compounds/calibrants with known experimental retention times [102].

As such, forward *in silico* library generation can prove to be a useful tool in the annotation of unknown structures where insufficient experimental data exists, allowing scientists to faster evaluate a larger identifiable space.

## Supplementary Information

Below is the link to the electronic supplementary material.

Supplementary file 1 (xlsx 16567 KB)

## Data Availability

The spectral libraries can be found at https://doi.org/10.5281/zenodo.14854025. All scripts used throughout this study can be found at https://github.com/eef-thepingvin/CFM-ID-forward-MS2-generation.

## Declarations

### Supplementary information

Supporting information is available free of charge. MZmine data processing workflow can be found in the Supporting Information (SI-5). All spectral matches from the NTA workflow (named using compound ID and m/z) have been collected as a.zip file and can be found at https://doi.org/10.5281/zenodo.14854025.

## References

[1] Manz KE, et al. Non-targeted analysis (NTA) and suspect screeninganalysis (SSA): a review of examining the chemical exposome. Journal of Exposure Science & Environmental Epidemiology. 2023;33(4):524–36. 37380877 10.1038/s41370-023-00574-6 PMC10403360

[2] Hao Z, et al. In: Chapter 13 - suspect and nontarget screening technologies for emerging contaminants (eds Liang, B., Gao, S.-H., Wang, H.-C. & Wang, A.-J.) water security: big data-driven risk identification, assessment and control of emerging contaminants 205–227 (Elsevier, 2024). https://www.sciencedirect.com/science/article/pii/B9780443141706000251.

[3] Paszkiewicz M, et al. Advances in suspect screening and non-target analysis of polar emerging contaminants in the environmental monitoring. TrAC, Trends Anal Chem. 2022;154:116671.

[4] Cui D, et al. Evaluating non-targeted analysis methods for chemical characterization of organic contaminants in different matrices to estimate children’s exposure. J Eposure Sci Environ Epidemiol. 2023;33(4):589–601. 10.1038/s41370-023-00547-9 PMC10148696 37120701

[5] Vosough M, Schmidt TC, Renner G. Non-target screening in water analysis: recent trends of data evaluation, quality assurance, and their future perspectives. Anal Bioanal Chem. 2024;416(9):2125–36. 38300263 10.1007/s00216-024-05153-8 PMC10951028

[6] Egede Frøkjær E, Rüsz Hansen H, Hansen M. Non-targeted and suspect screening analysis using ion exchange chromatography-Orbitrap tandem mass spectrometry reveals polar and very mobile xenobiotics in Danish drinking water. Chemosphere. 2023;339:139745. 37558003 10.1016/j.chemosphere.2023.139745

[7] Guy C, Duporte G, Luquot L, Gomez E. Non-target screening to track contaminant removal and release during nature-based water treatment. Frontiers in Environmental Science. 2024;12:1385806. https://hal.science/hal-04555006.

[8] Zhu L, et al. Suspect and non-target screening of chemicals of emerging arctic concern in biota, air and human serum. Environmental Pollution. 2024;360:124605. https://www.sciencedirect.com/science/article/pii/ S0269749124013198. 10.1016/j.envpol.2024.124605 39053798

[9] Aggerbeck MR, et al. Non-target analysis of Danish wastewater treatment plant effluent: statistical analysis of chemical fingerprinting as a step toward a future monitoring tool. Environmental Research. 2024;257:119242. https://www.sciencedirect.com/science/article/pii/S0013935124011472. 10.1016/j.envres.2024.119242 38821457

[10] Diera T, et al. A non-target screening study of high-density polyethylene pipes revealed rubber compounds as main contaminant in a drinking water distribution system. Water Res. 2023;229:119480. 10.1016/j.watres.2022.119480 36528929

[11] Nanusha MY, et al. Quantitative non-targeted screening to profile micropolutants in sewage sludge used for agricultural field amendments. Environmental Science & Technology. 2024;58:9850–62. 38758285 10.1021/acs.est.4c01441 PMC11155239

[12] Zhang P, et al. Defining the scope of exposome studies and research needs from a multidisciplinary perspective. 2021;8(10):839–52. 10.1021/acs.estlett.1c00648 PMC8515788 34660833

[13] Sdougkou K, et al. Longitudinal exposomics in a multiomic wellness cohort reveals distinctive and dynamic environmental chemical mixtures in blood. Environmental Science & Technology. 2024;58:16302–15. 39236221 10.1021/acs.est.4c05235 PMC11411717

[14] Hakme E, et al. Fate of pesticide residues in beer and its by-products. Food Additives and Contaminants - Part A. 2024;41(1):45–59. 10.1080/19440049.2023.2282557 38039344

[15] Vázquez Loureiro P, et al. Identification and quantification of per- and polyfluorinated alkyl substances (PFAS)migrating from food contact materials (FCM). Chemosphere. 2024;360: 142360. https://www.sciencedirect.com/science/article/pii/S0045653524012530. 10.1016/j.chemosphere.2024.142360 38761829

[16] Heinsvig PJ, Noble C, Dalsgaard PW, Mardal M. Forensic drug screening by liquid chromatography hyphenated with high-resolution mass spectrometry (LC-HRMS). TrAC, Trends Anal Chem. 2023;162:117023.

[17] Yang C, et al. Non-target screening analysis of hazardous noxious substances using gas chromatography-quadrupole time-of-flight mass spectrometry. Environmental Advances. 2024;18:100597. https://www.sciencedirect.com/science/article/pii/S2666765724001157.

[18] Bittremieux W, Wang M, Dorrestein PC. The critical role that spectral libraries play in capturing the metabolomics community knowledge. Metabolomics. 2022;18(12):94. 36409434 10.1007/s11306-022-01947-y PMC10284100

[19] Hollender J, et al. NORMAN guidance on suspect and non-target screening in environmental monitoring. Environ Sci Eur. 2023;35(1):75.

[20] Ruttkies C, Schymanski EL, Wolf S, Hollender J, Neumann S. MetFrag relaunched: incorporating strategies beyond in silico fragmentation. Journal of Cheminformatics. 2016;8(1):3. 26834843 10.1186/s13321-016-0115-9 PMC4732001

[21] Dührkop K, et al. SIRIUS 4: a rapid tool for turning tandem mass spectra into metabolite structure information. Nat Methods. 2019;16(4):299–302. 30886413 10.1038/s41592-019-0344-8

[22] Wang F, et al. CFM-ID 4.0: more accurate ESI-MS/MS spectral prediction and compound identification. Analytical Chemistry. 2021. 10.1021/acs.analchem.1c01465 PMC9064193 34403256

[23] Schymanski EL, et al. Identifying small molecules via high resolution mass spectrometry: communicating confidence. 2014. 10.1021/es5002105 24476540

[24] Black G, et al. Exploring chemical space in non-targeted analysis: a proposed ChemSpace tool. Anal Bioanal Chem. 2023;415(1):35–44. 36435841 10.1007/s00216-022-04434-4 PMC10010115

[25] Hulleman T, et al. Critical assessment of the chemical space covered by LC-HRMS non-targeted analysis. Environmental Science & Technology. 2023;57(38):14101–12. 37704971 10.1021/acs.est.3c03606 PMC10537454

[26] Samanipour S, et al. Exploring the chemical space of the exposome: how far have we gone? JACS Au. 2024;4:2412–25. 39055136 10.1021/jacsau.4c00220 PMC11267556

[27] Abrahamsson D, et al. In silico structure predictions for non-targeted analysis: from physicochemical properties to molecular structures. J Am Soc Mass Spectrom. 2022;33(7):1134–47. 35649165 10.1021/jasms.1c00386 PMC9365522

[28] Hupatz H, et al. Critical review on in silico methods for structural annotation of chemicals detected with LC/HRMS non-targeted screening. Anal Bioanal Chem. 2024;417(3):473–93. 39138659 10.1007/s00216-024-05471-x PMC11700063

[29] Mohammed Taha H, et al. The NORMAN Suspect List Exchange (NORMAN-SLE): facilitating European and worldwide collaboration on suspect screening in high resolution mass spectrometry. Environ Sci Eur. 2022;34(1):104. 36284750 10.1186/s12302-022-00680-6 PMC9587084

[30] Wank J, Redeker W, Taylor A, Herbold R, Lassahn P-G. High-resolution, accurate mass measurements and metabolite identification: an automated approach using fragment prediction in combination with Fragment Ion Search (FISH). Drug Metab Rev. 2011;43:10.

[31] Skinnider MA, et al. A deep generative model enables automated structure elucidation of novel psychoactive substances. Nature Machine Intelligence. 2021;3(11):973–84.

[32] Tsugawa H, et al. Hydrogen rearrangement rules: computational MS/MS fragmentation and structure elucidation using MS-FINDER software. Anal Chem. 2016;88(16):7946–58. 27419259 10.1021/acs.analchem.6b00770 PMC7063832

[33] Lai Z, et al. Identifying metabolites by integrating metabolome databases with mass spectrometry cheminformatics. Nat Methods. 2018;15(1):53–6. 29176591 10.1038/nmeth.4512 PMC6358022

[34] Dührkop K, Shen H, Meusel M, Rousu J, Böcker S. Searching molecular structure databases with tandem mass spectra using CSI:FingerID. Proceedings of the National Academy of Sciences of the United States of America. 2015. 10.1073/pnas.1509788112 PMC4611636 26392543

[35] Stravs MA, Dührkop K, Böcker S, Zamboni N. MSNovelist: de novo structure generation from mass spectra. Nat Methods. 2022;19(7):865–70. 35637304 10.1038/s41592-022-01486-3 PMC9262714

[36] Dulio V, et al. Beyond target chemicals: updating the NORMAN prioritisation scheme to support the EU chemicals strategy with semi-quantitative suspect/non-target screening data. Environmental Sciences Europe. 2024; 36. Publisher Copyright: The Author(s) 2024.

[37] Alygizakis NA, et al. NORMAN digital sample freezing platform: a European virtual platform to exchange liquid chromatography high resolution-mass spectrometry data and screen suspects in “digitally frozen” environmental samples. TrAC Trends in Analytical Chemistry. 2019;115:129–137 . https://www.sciencedirect.com/science/article/pii/S0165993619300354.

[38] Rostkowski P, et al. The strength in numbers: comprehensive characterization of house dust using complementary mass spectrometric techniques. Anal Bioanal Chem. 2019;411(10):1957–77. 10.1007/s00216-019-01615-6 PMC6458998 30830245

[39] Su QZ, Vera P, Nerín C. Combination of structure databases, in silico fragmentation, and MS/MS libraries for untargeted screening of non-volatile migrants from recycled high-density polyethylene milk bottles. Anal Chem. 2023;95(23):8780–8. 37262310 10.1021/acs.analchem.2c05389 PMC10267890

[40] Chao A, et al. In silico MS/MS spectra for identifying unknowns: a critical examination using CFM-ID algorithms and ENTACT mixture samples. Anal Bioanal Chem. 2020;412(6):1303–15. 31965249 10.1007/s00216-019-02351-7 PMC7021669

[41] Kind T, et al. LipidBlast in silico tandem mass spectrometry database for lipid identification. Nat Methods. 2013;10(8):755–8. 23817071 10.1038/nmeth.2551 PMC3731409

[42] Huan T, et al. MyCompoundID MS/MS search: metabolite identification using a library of predicted fragment-ion-spectra of 383,830 possible human metabolites. Anal Chem. 2015;87(20):10619–26. 26415007 10.1021/acs.analchem.5b03126

[43] Murphy M, et al. Efficiently predicting high resolution mass spectra with graph neural networks. 2023. arXiv:2301.11419.

[44] Wang F, et al. Deep learning-enabled MS/MS spectrum prediction facilitates automated identification of novel psychoactive substances. Analytical Chemistry Analytical Chemistry. 2023;95(50):18326–34. 38048435 10.1021/acs.analchem.3c02413 PMC10733899

[45] Zhu B, et al. Knowledge-based in silico fragmentation and annotation of mass spectra for natural products with MassKG. Computational and Structural Biotechnology Journal. 2024;23:3327–3341. https://www.sciencedirect.com/science/article/pii/S2001037024002897. 10.1016/j.csbj.2024.09.001 PMC11415640 39310281

[46] Perez-Ribera M, et al. SingleFrag: a deep learning tool for MS/MS fragment and spectral prediction and metabolite annotation. 2024. 10.1093/bib/bbaf333 PMC12245663 40641047

[47] Weininger D. SMILES, a chemical language and information system: 1: introduction to methodology and encoding rules. Journal of Chemical Information and Computer Sciences. 1988.

[48] Grulke CM, Williams AJ, Thillanadarajah I, Richard AM. EPA’s DSSTox database: history of development of a curated chemistry resource supporting computational toxicology research. Computational Toxicology. 2019;12:100096. 10.1016/j.comtox.2019.100096 PMC7787967 33426407

[49] McEachran AD, et al. Linking in silico MS/MS spectra with chemistry data to improve identification of unknowns. Scientific Data. 2019;6(1):141. 31375670 10.1038/s41597-019-0145-z PMC6677792

[50] Meekel N, Vughs D, Béen F, Brunner AM. Online prioritization of toxic compounds in water samples through intelligent HRMS data acquisition. Anal Chem. 2021;93(12):5071–80. 33724776 10.1021/acs.analchem.0c04473 PMC8153395

[51] Getzinger GJ, Higgins CP, Ferguson PL. Structure database and in silico spectral library for comprehensive suspect screening of per-and polyfluoroalkyl substances (PFASs) in environmental media by high-resolution mass spectrometry. Anal Chem. 2021;93(5):2820–7. 33496574 10.1021/acs.analchem.0c04109 PMC8011993

[52] Gallidabino MD, Hamdan L, Murphy B, Barron LP. Suspect screening of halogenated carboxylic acids in drinking water using ion exchange chromatography – high resolution (Orbitrap) mass spectrometry (IC-HRMS). Talanta. 2018;178:57–68. 29136864 10.1016/j.talanta.2017.08.092

[53] Lai A, et al. Retrospective non-target analysis to support regulatory water monitoring: from masses of interest to recommendations via in silico workflows. Environ Sci Eur. 2021;33(1):43.

[54] Aalizadeh R, et al. Development and application of liquid chromatographic retention time indices in HRMS-based suspect and nontarget screening. Anal Chem. 2021;93(33):11601–11. 34382770 10.1021/acs.analchem.1c02348

[55] Boelrijk J, van Herwerden D, Ensing B, Forré P, Samanipour S. Predicting RP-LC retention indices of structurally unknown chemicals from mass spectrometry data. Journal of Cheminformatics. 2023;15(1):28. 36829215 10.1186/s13321-023-00699-8 PMC9960388

[56] Fan Y, et al. Modelling and predicting liquid chromatography retention time for PFAS with no-code machine learning. Environmental Science: Advances. 2023;3(2):198–207.

[57] Souihi A, Mohai MP, Palm E, Malm L, Kruve A. MultiConditionRT: predicting liquid chromatography retention time for emerging contaminants for a wide range of eluent compositions and stationary phases. J Chromatogr A. 2022;1666:462867. 35139450 10.1016/j.chroma.2022.462867

[58] Bonini P, Kind T, Tsugawa H, Barupal DK, Fiehn O. Retip: retention time prediction for compound annotation in untargeted metabolomics. Anal Chem. 2020;92(11):7515–22. 32390414 10.1021/acs.analchem.9b05765 PMC8715951

[59] Schmid R, et al. Integrative analysis of multimodal mass spectrometry data in MZmine 3. Nat Biotechnol. 2023;41(4):447–9. 36859716 10.1038/s41587-023-01690-2 PMC10496610

[60] Tsugawa H, et al. MS-DIAL: data-independent MS/MS deconvolution for comprehensive metabolome analysis. Nat Methods. 2015;12(6):523–6. 25938372 10.1038/nmeth.3393 PMC4449330

[61] Allen F, Pon A, Wilson M, Greiner R, Wishart D. CFM-ID: a web server for annotation, spectrum prediction and metabolite identification from tandem mass spectra. Nucleic Acids Res. 2014;42(W1):W94–9. 24895432 10.1093/nar/gku436 PMC4086103

[62] Allen F, Greiner R, Wishart D. Competitive fragmentation modeling of ESI-MS/MS spectra for putative metabolite identification. Metabolomics. 2015.

[63] Allen F, Pon A, Greiner R, Wishart D. Computational prediction of electron ionization mass spectra to assist in GC/MS compound identification. Anal Chem. 2016;88(15):7689–97. 27381172 10.1021/acs.analchem.6b01622

[64] Djoumbou-Feunang Y, et al. CFM-ID 3.0: significantly improved ESI-MS/MS prediction and compound identification. Metabolites. 2019;9(4):72. 10.3390/metabo9040072 PMC6523630 31013937

[65] Wang F, et al. CFM-ID 4.0 - a web server for accurate MS-based metabolite identification. Nucleic Acids Research. 2022. 10.1093/nar/gkac383 PMC9252813 35610037

[66] Bezanson J, Karpinski S, Shah VB, Edelman A. Julia: a fast dynamic language for technical computing. CoRR abs/1209.5145. 2012. http://arxiv.org/abs/1209.5145.

[67] Rdkit: Open-source cheminformatics. https://www.rdkit.org.

[68] Kim S, Thiessen PA, Cheng T, Yu B, Bolton EE. An update on PUG-REST: RESTful interface for programmatic access to PubChem. Nucleic Acids Res. 2018;46(W1):W563–70. 29718389 10.1093/nar/gky294 PMC6030920

[69] Inc. TM. Matlab version: 24.1.0.2689473 (r2024a). 2024. https://www.mathworks.com.

[70] Inc. TM. Database explorer version: 24.1 (r2024a). 2024. https://www.mathworks.com.

[71] Kim S, et al. PubChem 2023 update. Nucleic Acids Res. 2023;51(D1):D1373–80. 36305812 10.1093/nar/gkac956 PMC9825602

[72] Pence HE, Williams A. Chemspider: an online chemical information resource. 2010.

[73] Nanusha MY, et al. Explorative quantitative nontarget analysis reveals micropollutants in Danish groundwater. ACS ES and T Water. 2023;3(12):3992–4003.

[74] Cleveland WS, Devlin SJ. Locally weighted regression: an approach to regression analysis by local fitting. J Am Stat Assoc. 1988;83(403):596–610.

[75] Schmid R, et al. Ion identity molecular networking for mass spectrometry-based metabolomics in the GNPS environment. Nat Commun. 2021;12(1):3832. 34158495 10.1038/s41467-021-23953-9 PMC8219731

[76] Brungs C, et al. Efficient generation of open multi-stage fragmentation mass spectral libraries. 2024. 10.1038/s41592-025-02813-0 PMC12510872 40954295

[77] Horai H, et al. MassBank: a public repository for sharing mass spectral data for life sciences. J Mass Spectrom. 2010;45(7):703–14. 20623627 10.1002/jms.1777

[78] Wang M, et al. Sharing and community curation of mass spectrometry data with Global Natural Products Social Molecular Networking. Nat Biotechnol. 2016;34(8):828–37. 27504778 10.1038/nbt.3597 PMC5321674

[79] Mardal M, et al. HighResNPS.com: an online crowd-sourced HR-MS database for suspect and non-targeted screening of new psychoactive substances. Journal of Analytical Toxicology. 2019;43(7):520-527. 10.1093/jat/bkz030 31095696

[80] Folberth J, Begemann K, Jöhren O, Schwaninger M, Othman A. MS2 and LC libraries for untargeted metabolomics: enhancing method development and identification confidence. J Chromatogr, B: Anal Technol Biomed Life Sci. 2020;1145:122105. 10.1016/j.jchromb.2020.122105 32305706

[81] Wang X, et al. Machine learning–enhanced molecular network reveals global exposure to hundreds of unknown PFAS. Science Advances. 2024;10:eadn1039. https://www.science.org/doi/abs/10.1126/sciadv.adn1039. 10.1126/sciadv.adn1039 PMC11114235 38781329

[82] Szabó D, Schlosser G, Vékey K, Drahos L, Révész Á. Collision energies on QTof and Orbitrap instruments: how to make proteomics measurements comparable? J Mass Spectrom. 2021;56(1):e4693. 33277714 10.1002/jms.4693

[83] Nanusha MY, et al. Unravelling the occurrence of trace contaminants in surface waters using semi-quantitative suspected non-target screening analyses. Environ Pollut. 2022;315:120346. 36202272 10.1016/j.envpol.2022.120346

[84] Nanusha MY, et al. Nontarget analysis of drinking water from Danish waterworks: profiling of organic micropollutants and health risk screening. ACS ES &T Water. 2025;0:null.

[85] Fukuhara N, Bigelow LA. The action of elementary fluorine upon organic compounds. X. The vapor phase fluorination of acetone. Journal of the American Chemical Society. 1941;63(3):788-791.

[86] Lemal DM. Perspective on fluorocarbon chemistry. J Org Chem. 2004;69(1):1–11. 14703372 10.1021/jo0302556

[87] Bittremieux W, et al. Comparison of cosine, modified cosine, and neutral loss based spectrum alignment for discovery of structurally related molecules. J Am Soc Mass Spectrom. 2022;33(9):1733–44. 10.1021/jasms.2c00153 35960544

[88] Alygizakis N, et al. Towards a harmonized identification scoring system in LC-HRMS/MS based non-target screening (NTS) of emerging contaminants. TrAC - Trends in Analytical Chemistry. 2023;159:116944.

[89] Kubilius DT, Bushway RJ. Determination of hexazinone and its metabolites in groundwater by capillary electrophoresis. J Chromatogr A. 1998;793(2):349–55. 9474788 10.1016/s0021-9673(97)00913-8

[90] Gadaleta D, Lombardo A, Toma C, Benfenati E. A new semi-automated workflow for chemical data retrieval and quality checking for modeling applications. Journal of Cheminformatics. 2018;10(1):60. 30536051 10.1186/s13321-018-0315-6 PMC6503381

[91] Jonge N, et al. Reproducible MS/MS library cleaning pipeline in matchms. Journal of Cheminformatics. 2024;16:88. 10.1186/s13321-024-00878-1 PMC11285329 39075613

[92] Varga E, et al. Development and validation of a (semi-)quantitative UHPLC-MS/MS method for the determination of 191 mycotoxins and other fungal metabolites in almonds, hazelnuts, peanuts and pistachios. Anal Bioanal Chem. 2013;405(15):5087–104. 23471368 10.1007/s00216-013-6831-3 PMC3656230

[93] Tsao YC, Lai YC, Liu HC, Liu RH, Lin DL. Simultaneous determination and quantitation of paraquat, diquat, glufosinate and glyphosate in postmortem blood and urine by LC-MS-MS. J Anal Toxicol. 2016;40(6):427–36. 27339477 10.1093/jat/bkw042

[94] Cui L, Nithipatikom K, Campbell WB. Simultaneous analysis of angiotensin peptides by LC-MS and LC-MS/MS: metabolism by bovine adrenal endothelial cells. Anal Biochem. 2007;369(1):27–33. 17681269 10.1016/j.ab.2007.06.045 PMC2754136

[95] Djoumbou-Feunang Y, et al. BioTransformer: a comprehensive computational tool for small molecule metabolism prediction and metabolite identification. Journal of Cheminformatics. 2019;11(1):2. 30612223 10.1186/s13321-018-0324-5 PMC6689873

[96] Wishart DS, et al. BioTransformer 3.0 - a web server for accurately predicting metabolic transformation products. Nucleic Acids Research. 2022. 10.1093/nar/gkac313 PMC9252798 35536252

[97] Wicker J, et al. enviPath - the environmental contaminant biotransformation pathway resource. Nucleic Acids Research. 2016. 10.1093/nar/gkv1229 PMC4702869 26582924

[98] Hafner J, et al. Advancements in biotransformation pathway prediction: enhancements, datasets, and novel functionalities in enviPath. Journal of Cheminformatics. 2024;16:93. 39107805 10.1186/s13321-024-00881-6 PMC11304562

[99] Giera M, Aisporna A, Uritboonthai W, Siuzdak G. The hidden impact of in-source fragmentation in metabolic and chemical mass spectrometry data interpretation. Nat Metab. 2024;6:1647–8. 38918534 10.1038/s42255-024-01076-x PMC11826480

[100] Guo J, Shen S, Xing S, Yu H, Huan T. ISFrag: de novo recognition of in-source fragments for liquid chromatography-mass spectrometry data. Anal Chem. 2021;93(29):10243–50. 34270210 10.1021/acs.analchem.1c01644

[101] Stanstrup J, Neumann S, Vrhovšek U. PredRet: prediction of retention time by direct mapping between multiple chromatographic systems. Anal Chem. 2015;87(18):9421–8. 26289378 10.1021/acs.analchem.5b02287

[102] van Herwerden D. Computational non-targeted analysis strategies for exploring the exposome chemical space. Phd thesis, University of Amsterdam, Van ’t Hoff Institute for Molecular Sciences. 2024. Available at https://hdl.handle.net/11245.1/0fdf0846-faba-4b37-ba87-282c333a7c52.

